# Evaluation of Body Mass Index, Overweight and Obesity Status, and Cholesterol Levels in Younger Children

**DOI:** 10.1001/jamanetworkopen.2023.8141

**Published:** 2023-04-13

**Authors:** Ursa Sustar, Olga Kordonouri, Stefan Arens, Jernej Kovac, Katarina Sedej, Tadej Battelino, Urh Groselj

**Affiliations:** 1 Department of Endocrinology, Diabetes, and Metabolic Diseases, University Children’s Hospital, UMC Ljubljana, Ljubljana, Slovenia; 2 Faculty of Medicine, University of Ljubljana, Ljubljana, Slovenia; 3 Children’s Hospital Auf der Bult, Hannover, Germany; 4 Clinical Institute of Special Laboratory Diagnostics, University Children’s Hospital, University Medical Centre Ljubljana, Ljubljana, Slovenia; 5 Community Health Centre Ljubljana, Unit Siska, Ljubljana, Slovenia

## Abstract

This cohort study examines cholesterol levels in children with overweight or obesity.

## Introduction

Overweight and obesity are public health concerns associated with dyslipidemia and cardiovascular disease risk factors.^1^ Childhood dyslipidemia results either from inherited or nongenetic factors.^2^ We aimed to assess the association between overweight and obesity status and cholesterol levels in a cohort of younger children from 2 independent populations.

## Methods

Data on weight, height, and low-density lipoprotein cholesterol (LDL-C) and total cholesterol (TC) levels were collected as part of regular medical examinations of children aged 2 to 6 years. A total of 12 111 children from the German (born 2011-2017) cohort and 3535 children from the Slovenian (born 2007-2010) cohort were included. The study was conducted from January 6, 2020, to March 9, 2022. Descriptive statistics (median [IQR]) were used to analyze the demographic and clinical data of participants with complete data. Linear regression was used to study the association between LDL-C and TC level and body mass index standard deviation score (BMI SDS). Analysis of LDL-C and TC levels and BMI SDS was performed with the Pearson correlation coefficient. The BMI SDS values in children were ranked: severely obese (≥99th percentile), obese (≥95th-99th percentile), overweight (≥85th-95th percentile), normal (≥5th-85th percentile), and underweight (<5th percentile). Wilcoxon signed-rank test was used for comparing groups by LDL-C and TC levels. A false discovery rate–adjusted *P* value was calculated for multiple comparison corrections of the 2-sided, unpaired *P* value, with significance at *P* < .05. Statistical analysis was performed with R, version 4.2.1 (R Foundation for Statistical Computing). The study was approved by the Slovenian National Medical Ethics Committee and the Ethics Committee of Hannover Medical School. In the German cohort, written consent of at least 1 parent or other primary caregiver was obtained; consent was not needed in the Slovenian cohort because TC measurement is a part of the regular examination of children aged 5 years. This study followed the STROBE reporting guideline.

## Results

Demographic and clinical characteristics of the children are presented in the Table. The LDL-C level in children aged 4.0 (IQR, 3.0-5.1) years was 92.7 (IQR, 77.2-108.1) mg/dL and TC level in children aged 5.1 (IQR, 5.0-5.2) years was 158.3 (IQR, 142.9-177.6) (to convert to millimoles per liter, multiply by 0.0259). A weak correlation between LDL-C and TC levels and BMI SDS was observed in the German (*R* = 0.037; *P* < .001) and Slovenian (*R* = 0.052; *P* = .002) cohorts (Figure, A and B). Nonsignficant negative correlations were observed for height. Figure, C and D present differences between children according to their BMI SDS category.

**Table.** Demographic and Clinical Characteristics of the Children From the German and Slovenian Cohorts by Weight Category

Variable	Median (IQR)				*P* value
	Underweight	Normal	Overweight and obese	Total	
**German cohort**					
No. (%)	423 (3.5)	9525 (78.6)	2163 (17.9)	12 111	
Age, y	3.9 (2.9 to 5.1)	4.0 (3.0 to 5.1)	3.8 (2.9 to 5.0)	4.0 (3.0 to 5.1)	<.001
BMI SDS	−2.1 (−2.5 to −1.8)	0.0 (−0.5 to 0.5)	1.5 (1.2 to 1.9)	0.2 (−0.4 to 0.8)	<.001
LDL-C, mg/dL	88.8 (73.4 to 104.2)	92.7 (77.2 to 108.1)	92.7 (81.1 to 108.1)	92.7 (77.2 to 108.1)	.001
Sex, No. (%)				y	
Female	190 (44.9)	4564 (47.9)	1028 (47.5)	5782 (47.7)	.47
Male	233 (55.1)	4961 (52.1)	1135 (52.5)	6329 (52.3)	
**Slovenian cohort**					
No. (%)	167 (4.7)	2866 (81.1)	502 (14.2)	3535	
Age, y	5.1 (5.0 to 5.2)	5.1 (5.0 to 5.2)	5.1 (5.0 to 5.2)	5.1 (5.0 to 5.2)	.75
BMI SDS	−2.0 (−2.3 to −1.8)	−0.1 (−0.7 to 0.3)	1.5 (1.2 to 1.9)	−0.0 (−0.7 to 0.6)	<.001
TC, mg/dL	158.3 (146.7 to 177.6)	158.3 (142.9 to 177.6)	158.3 (142.9 to 177.6)	158.3 (142.9 to 177.6)	.02
Sex, No. (%)					
Female	85 (50.9)	1373 (47.9)	244 (48.6)	1702 (48.1)	.74
Male	82 (49.1)	1493 (52.1)	258 (51.4)	1833 (51.9)	

Abbreviations: BMI SDS, body mass index standard deviation score; LDL-C, low-density lipoprotein cholesterol; TC, total cholesterol.

SI conversion: To convert LDL-C and TC to millimoles per liter, multiply by 0.0259.

**Figure.**
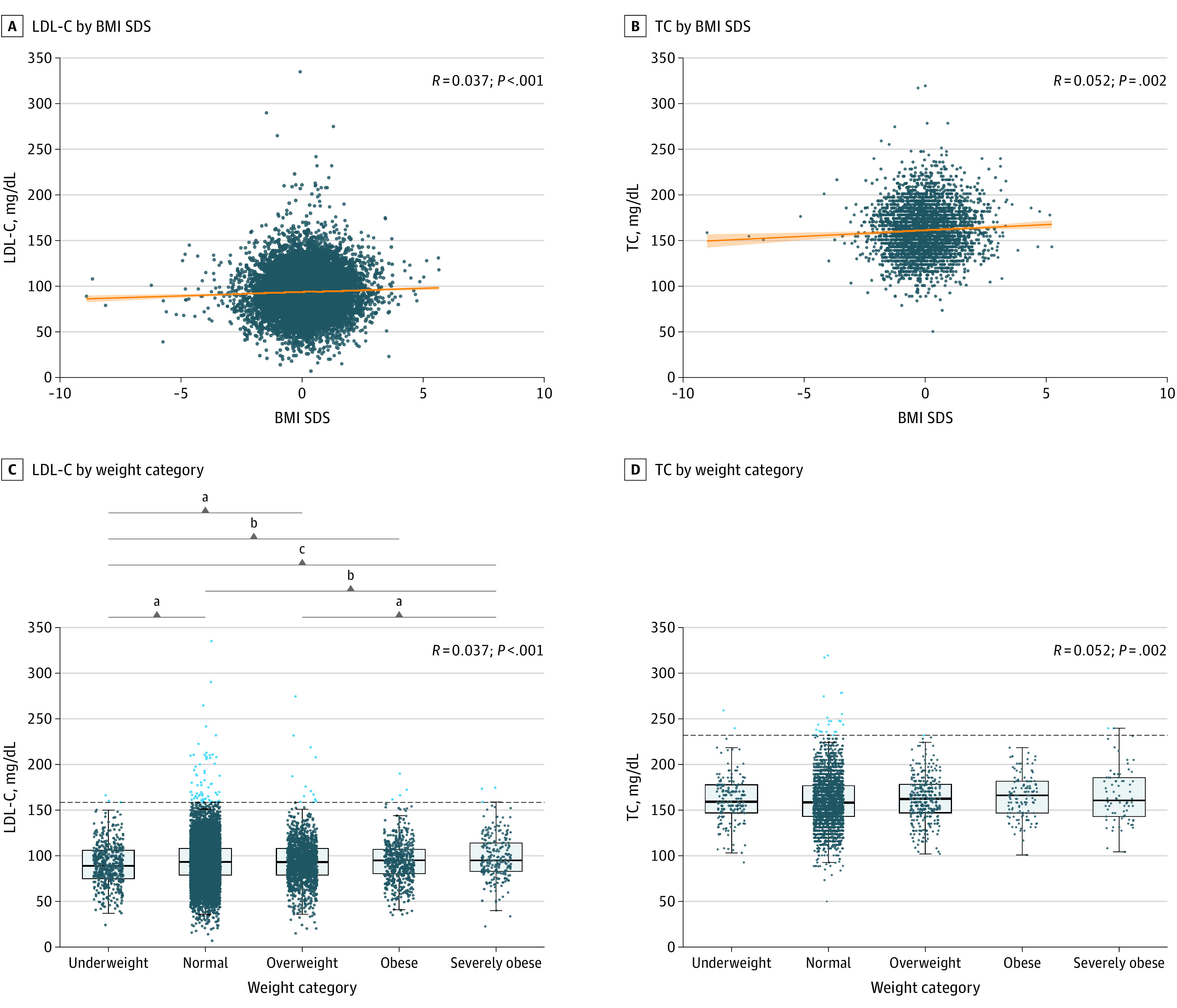
Low-Density Lipoprotein Cholesterol (LDL-C) and Total Cholesterol (TC) Levels According to the Body Mass Index Standard Deviation Score (BMI SDS) in the General Pediatric Population Association between LDL-C (A) and TC (B) levels and BMI SDS of the general pediatric population. Pairwise comparison of the LDL-C (C) and TC (D) levels of the children categorized according to their BMI SDS: severely obese (>99th percentile), obese (95th-99th percentile), overweight (85th-95th percentile), normal (5th-85th percentile), and underweight (<5th percentile). Extreme levels of LDL-C (>158.3 mg/dL) and TC (>213.7 mg/dL) (to convert to millimoles per liter, multiply by 0.0259) are represented with dots. The dashed line represents the cutoff level for LDL-C and TC. ^a^*P* < .05. ^b^*P* < .01. ^c^*P* < .001.

## Discussion

Children with overweight and obesity with dyslipidemia^3^ and children with familial hypercholesterolemia (FH)^4,5^ are at risk of developing cardiovascular diseases. In both cohorts, cholesterol levels were weakly correlated with an increasing BMI SDS. Severely obese children had a modest increase in LDL-C levels compared with children from other BMI SDS categories. This was not observed with TC levels, suggesting that children with obesity had reduced levels of high-density lipoprotein cholesterol.^6^ Our results support that an indication for FH due to severe increases in TC or LDL-C levels was present primarily in children with normal weight.

One study limitation is that the screening approaches of both cohorts were not completely comparable. In the German cohort, the major limitation was the optional participation in the LDL-C level measurement, which may result in a potential bias toward inclusion of families with increased cardiovascular disease risk or FH or obesity predisposition.

The findings of this study suggest that higher BMI SDS and/or overweight and obesity status do not contribute to substantially elevated cholesterol levels in younger children. Mildly increased LDL-C levels were associated with higher BMI SDS or severe obesity at the population level but were found to be clinically unimportant at the individual level. This suggests a relatively larger role of genetic factors compared with environmental factors in cholesterol levels in younger children.

## Notes

Supplement.
Data Sharing Statement

## References

[1] Umer A, Kelley GA, Cottrell LE, Giacobbi P Jr, Innes KE, Lilly CL. Childhood obesity and adult cardiovascular disease risk factors: a systematic review with meta-analysis. BMC Public Health. 2017;17(1):683. doi:10.1186/s12889-017-4691-z 28851330 PMC5575877

[2] Nordestgaard BG, Chapman MJ, Humphries SE, ; European Atherosclerosis Society Consensus Panel. Familial hypercholesterolaemia is underdiagnosed and undertreated in the general population: guidance for clinicians to prevent coronary heart disease: consensus statement of the European Atherosclerosis Society. Eur Heart J. 2013;34(45):3478-90a. doi:10.1093/eurheartj/eht273 23956253 PMC3844152

[3] Lavie CJ, Arena R, Alpert MA, Milani RV, Ventura HO. Management of cardiovascular diseases in patients with obesity. Nat Rev Cardiol. 2018;15(1):45-56. doi:10.1038/nrcardio.2017.108 28748957

[4] Groselj U, Wiegman A, Gidding SS. Screening in children for familial hypercholesterolaemia: start now. Eur Heart J. 2022;43(34):3209-3212. doi:10.1093/eurheartj/ehac224 35511818

[5] Sustar U, Kordonouri O, Mlinaric M, . Universal screening for familial hypercholesterolemia in 2 populations. Genet Med. 2022;24(10):2103-2111. doi:10.1016/j.gim.2022.06.010 35913489

[6] Bora K, Pathak MS, Borah P, Das D. Association of decreased high-density lipoprotein cholesterol (HDL-C) with obesity and risk estimates for decreased HDL-C attributable to obesity: preliminary findings from a hospital-based study in a city from northeast India. J Prim Care Community Health. 2017;8(1):26-30. doi:10.1177/2150131916664706 27531078 PMC5932653

