# Supplementary material for: Evaluation of Body Mass Index, Overweight and Obesity Status, and Cholesterol Levels in Younger Children
Source: JAMA Netw Open. 2023 Apr 13;6(4):e238141. doi: 10.1001/jamanetworkopen.2023.8141 (PMC10102874; doi:10.1001/jamanetworkopen.2023.8141)
Supplement: Supplement. — Data Sharing Statement [file jamanetwopen-e238141-s001.pdf]

Data Sharing Statement

Sustar. Evaluation of Body Mass Index, Overweight and Obesity Status, and Cholesterol Levels in Younger Children. *JAMA Netw Open*. Published April 13, 2023. doi:10.1001/jamanetworkopen.2023.8141

Data
Data available: Yes
Data types: Deidentified participant data
How to access data: urh.groselj@kclj.si
When available: With publication

Supporting Documents
Document types: None

Additional Information
Who can access the data: Data will be made available upon reasonable request.
Types of analyses: Data will be made available for a specified purpose.
Mechanisms of data availability: Data will be made available after approval of a proposal.
Any additional restrictions: Ethical approval.
